# Editorial Changes and New Publications

**Published:** 1854-06

**Authors:** 


EDITORIAL CHANGES AND NEW PUBLICATIONS.

Dr. Joseph Parrish has removed to Philadelphia and has resigned the editorial charge of the *New Jersey Medical Reporter* into the hands of Dr. Butler, who is regarded as eminently qualified for his new position. The *Stethoscope* has also changed hands and is now edited by a committee of the Medical Society of Virginia. The *American Medical Monthly* is a new "star" in the horizon of medical literature and it shines with much brightness. It is edited by Dr. Parker, formerly of the N. Hampshire Journal of Medicine. It deserves success and if it be equal to its merits, justice will be awarded to the deserving. We observe that a new medical journal has been established at San Francisco, California. Drs. Phinney and Angle are the editors. We know not its title, but would suggest that of "The Aurum," an indispensible element in the success of all journals. Would be gratified to peruse Dr. Peaslee's monograph on the fœtal circulation.

☞ We have received through the mail, from that great fount of medical literature, Blanchard & Lea, of Phila., Bennet's work on "Pulmonary Consumption" and "La Roche on Pneumonia," notices of which will appear hereafter. Our medical friends who desire recent and approved works can make arrangements through the mail for small books, or by the express for works of any size. We will cheerfully aid in the procurement of those which may be required and which are not usually kept in any of our book stores. Complaint has been repeatedly made to us, by medical men of the State, that they were denied the opportunity of keeping pace with the science, because of the difficulty in the procurement of recent and valuable contributions. This we hope will soon be done away, as Mr. Ogden, of our City Book Store, intends shortly to supply himself with a good assortment of standard medical works.

Eastern publishers, we opine, have labored under an error in supposing that the profession of this State is but limited in numbers, and that there is therefore but little demand for fresh medical literature. They have therefore not regarded this as a desirable field for occupation, and yet we are assured that there is no where to be found a more anxious spirit of enquiry than among the medical men of this State, and we are sure too that no body of men have made more sacrifices to procure good scientific works, than our medical brethren here.—When Prof. Ford was in N. York, the publishing house of "Woods" informed him that several publications had been forwarded by mail to us. This surprised us somewhat, as they have not been received. In the haste of distribution in the P. O., these *may* have taken a wrong direction, for we have several times found in our box, documents not belonging to us, which we have always returned. 'Tis very strange that no stray document has EVER been returned to us. We would again ask our exchanges and all others to mark documents, "Box 109," in plain figures.

